# Protein profile of leprosy patients with plantar ulcers from the Eastern Amazon region

**DOI:** 10.1186/s40249-017-0318-y

**Published:** 2017-09-04

**Authors:** Marineia Porto de Oliveira, Jorge Rodrigues de Sousa, Rafael Silva de Araujo, Tinara Leila de Sousa Aarão, Juarez Antonio Simões Quaresma

**Affiliations:** 1 Nucleo de Medicina Tropical, Universidade Federal do Para, Av. Generalissimo Deodoro 92, Umarizal, Belem, Para 66055240 Brazil; 2 Centro de Ciencias Biologicas e da Saude, Universidade do Estado do Para, Belem, Para Brazil

**Keywords:** Leprosy, Plantar ulcer, Nutritional status, Anthropometric assessment, Albumin, Transferrin, C-reactive protein

## Abstract

**Background:**

Studies investigating the nutritional status of patients with leprosy and plantar ulcers are sparse. Therefore, the objective of this study was to describe the protein profile of leprosy patients with plantar ulcers from the Eastern Amazon region.

**Methods:**

A case record form was created for 75 patients with leprosy (31 with plantar ulcers and 44 without plantar ulcers) with the following data: sociodemographic characteristics, clinical form of leprosy, presence or absence of plantar ulcers, and nutritional assessment using anthropometry consisting of the measurement of body mass index, arm circumference, arm muscle circumference, and triceps skinfold. Levels of blood albumin, transferrin, and C-reactive protein (CRP) were also measured. Data regarding protein intake were obtained using a Food Frequency Questionnaire.

**Results:**

Plantar ulcers occurred more frequently in male patients (67.7%), patients aged 40–49 years (mean ± *SD*: 47.3 ± 8.0 years), and patients receiving 300 or 600 USD (71.0%). The mean weight and height of patients were 71.6 ± 11.4 kg and 1.62 ± 0.1 m, respectively. High levels of CRP were detected in 51.6% of leprosy patients with plantar ulcers and only 9.1% of patients without plantar ulcers (*P* < 0.001). Nutritional depletion of transferrin was observed in 14.3% of patients with paucibacillary leprosy and 44.3% of patients with multibacillary leprosy (*P* = 0.0447). Most patients had normal levels of serum albumin (74.2% with plantar ulcers and 77.3% without plantar ulcers).

**Conclusions:**

Most leprosy patients with plantar ulcers have normal levels of serum albumin and transferrin and high CRP levels, which indicates the presence of an inflammatory process. Our findings suggest the need to monitor patients with leprosy to prevent the occurrence of plantar ulcers and to provide adequate treatment for patients with existing plantar ulcers.

**Electronic supplementary material:**

The online version of this article (doi:10.1186/s40249-017-0318-y) contains supplementary material, which is available to authorized users.

## Multilingual abstracts

Please see Additional file 1 for translations of the abstract into the five official working languages of the United Nations.

## Background

The World Health Organization (WHO) defines leprosy as a public health problem, particularly in countries where the prevalence of the disease exceeds one case per 10,000 inhabitants [1]. About 47,000 new cases are detected each year in Brazil [2, 3]. Skin ulcers, such as those seen in patients with leprosy, are a serious public health problem. Infected chronic ulcers can lead to osteomyelitis and amputation of the affected limbs. These sequelae can have a negative impact on the family, social, and professional lives of affected individuals, who also often face financial difficulties [4]. Furthermore, the well-being and self-esteem of these individuals may be diminished by pain, loss of the ability to walk, and loss of independence. The appearance and unpleasant odour of the lesions may lead to social isolation [5].

Most lesions in patients with leprosy are located on the lower limbs [6]. Lower-extremity ulcers in patients with leprosy patients can be divided into two categories: leprous ulcers (due to the disease itself) and neuropathic ulcers (due to nervous system involvement) [6]. Plantar ulcers can cause a loss of protective sensitivity or lack of sensation in the plantar region from damage to the tibial nerve [6]. Other factors increase the risk of developing plantar ulcers, including paralysis, volume loss of intrinsic foot muscles, loss of the fat pad under the metatarsal head, anhidrotic skin, biomechanical alterations, and/or deformities (e.g., foot drop, structural bone alterations). Lengthy walks, long strides, and running, also contribute to the development of plantar ulcers [7].

An inflammatory process triggers mechanisms called acute-phase responses, which are characterized by a reduction in the levels of transport proteins such as albumin and transferrin (also known as negative acute-phase proteins) and an increase in the levels of positive acute-phase proteins such as C-reactive protein (CRP). CRP participates in the defence mechanisms of an organism. These proteins, which are synthesized by the liver, function as transport proteins in a variety of metabolic processes. They can be used to identify patients who are at risk of developing malnutrition before the disease becomes chronic, as an indirect relationship between these proteins and nutritional status has been reported [8].

There is a lack of studies evaluating the nutritional status of patients with lower-extremity ulcers. Therefore, we aimed to investigate the protein profile of leprosy patients with plantar ulcers in order to prevent these deficiencies through early nutritional intervention, in conjunction with a balanced diet and adequate protein intake for the healing of the lesions.

## Methods

### Study population and location

The participants were 75 adults with leprosy who registered and were treated at the outpatient clinic of the Demétrio Medrado Specialized Referral Unit (Unidade de Referência Especializada Demétrio Medrado; URE), State Department of Public Health, Belém, Pará, Eastern Amazon region, Brazil. The participants were divided into two groups: group 1 consisted of 31 leprosy patients with plantar ulcers, whereas group 2 consisted of 44 leprosy patients without plantar ulcers.

### Inclusion and exclusion criteria

The study population included patients of both sexes who were 20 to 60 years of age. Patients were required to have a diagnosis of leprosy confirmed by clinical features and bacilloscopic and/or histopathological examination [9], to be registered and receiving regular dressings at the URE, and to have agreed to participate in the study by signing an informed consent statement. We excluded patients who had no diagnosis of leprosy; patients who were younger than 20 years or older than 60 years of age; and patients with liver disease, oedema, and/or diabetes mellitus.

### Data collection

Data were collected over a period of 6 months using a case record form that contained the following data: identification, sociodemographic characteristics, clinical form of leprosy, presence or absence of plantar ulcers, and nutritional assessment by anthropometry for the measurement of body mass index (BMI), arm circumference (AC), arm muscle circumference (AMC), and triceps skinfold (TSF). Biochemical parameters (serum albumin, transferrin, and CRP) were also collected. Data regarding protein intake were obtained using a food frequency questionnaire.

### Anthropometric parameters

#### Body mass index

BMI was calculated as a patient’s current weight (kg) divided by the square of the patient’s height (m^2^). The criteria proposed by the WHO [10] were used for nutritional classification of the subjects. Current weight was measured as described by Dumler and Kilates [11]. Height was measured according to the method of Kennedy and Guthrie [12].

#### Arm circumference

Arm circumference is the sum of bone, muscle, and fat areas [13]. In this study, AC was measured to the nearest 0.5 cm using an inelastic measuring tape calibrated in millimetres, as described by Dumler and Kilates [11]. The results were interpreted according to Frisancho [14] and were classified using the reference values of Blackburn and Thornton [15].

#### Arm muscle circumference

Arm muscle circumference is a measure of the muscle reserves without correction for bone mass [13]. This parameter was obtained with a mathematical equation that uses AC and TSF. The results were interpreted according to the normal range proposed by Frisancho [16] and were classified using the reference values adapted from Blackburn and Thornton [15].

#### Triceps skinfold thickness

Skinfold thickness can be used to estimate body fat reserves. We measured the TSF with a Lange caliper according to the method of Dumler and Kilates [11]. The results were interpreted according to Frisancho [14] and classified using the reference values of Blackburn and Thornton [15].

### Laboratory analysis

The laboratory tests were carried out at the Laboratory of Clinical Analysis of the Center of Tropical Medicine, Federal University of Para (Nucleo de Medicina Tropical da Universidade Federal do Para; NMT/UFPA). The patients were asked to fast for 12 h before blood collection. The blood samples were centrifuged at 3500 rpm for 10 min to separate the serum. The serum concentrations of albumin, transferrin, and CRP were analysed for our study.

Total protein, albumin, transferrin, and CRP were assayed by spectrophotometry (semi-automatic TP Analyzer Plus, Thermoplate). Albumin was measured using a colorimetric method according to the manufacturer’s instructions (Doles). Transferrin was measured by immunoturbidimetry according to the manufacturer’s recommendations (Biotecnica). CRP was assayed by latex agglutination according to the manufacturer’s recommendations (Doles).

The albumin and transferrin results were compared to the reference values proposed by Bottoni [17]. The CRP results were compared to the reference values from the Laboratory of Clinical Analysis of NMT/UFPA.

### Food intake

A semi-quantitative Food Frequency Questionnaire was used in our study. The questionnaire consisted of a structured list of foods, in addition to different frequency categories.

A method adapted from Sichieri [18] was used to analyse the patients’ food profiles. The consumption rate was calculated by transforming the reported frequencies into fractions of daily frequency. The weighted mean of the consumption rate was calculated and the following cut-off values were used to categorize the intake: < 0.33 for low food intake, 0.34–0.65 for medium food intake, and ≥0.66 for high food intake.

### Clinical classification of leprosy

The WHO classification of leprosy was used in this study [19]. Patients were classified into two types according to the number of skin lesions and nerves involved: paucibacillary (PB) leprosy for 2–5 skin lesions, and multibacillary (MB) leprosy for more than 5 skin lesions.

### Statistical analysis

Data were stored in a database constructed using the BioEstat 5.0 program [20]. The frequencies and means of the variables were compared and differences were analysed using the chi-square test, Fisher’s exact test, and G-test. The level of significance was set at 5% (*P* ≤ 0.05) for our analyses.

## Results

### Sociodemographic variables

The basic characteristics of leprosy patients with and without plantar ulcers are shown in Table 1.

**Table 1:** Distribution of patients according to sociodemographic variables and the presence or absence of plantar ulcer

Parameters	With ulcer (*n* = 31)		No ulcer (*n* = 44)	
	*n*	(%)	*n*	(%)
Gender				
Male	21	(67.7)	27	(61.4)
Female	10	(32.3)	17	(38.6)
Age (years)				
20–29	02	(6.5)	01	(2.3)
30–39	06	(19.4)	11	(25.0)
40–49	12	(38.7)	10	(22.7)
50–60	11	(35.5)	22	(50.0)
Matrial status				
Married	18	(58.1)	31	(70.5)
Single	13	(41.9)	13	(29.5)
Profession				
None	29	(93.5)	44	(100.0)
Teacher	02	(6.5)	-	-
Occupation				
Retirement/Benefits	16	(51.6)	13	(29.5)
Housekeeper	05	(16.1)	08	(18.2)
Bricklayer	02	(6.5)	03	(6.8)
Others	08	(25.8)	20	(45.5)
Famaly income				
< 1 Minimum wage	08	(25.8)	14	(31.8)
1–2 Minimum wage	22	(71.0)	24	(54.5)
> 2 Minimum wage	01	(3.2)	06	(13.6)
Level of education				
Illiterate	04	(12.9)	02	(4.5)
Literate	01	(3.2)	-	-
Primary incomplete	17	(54.8)	26	(59.1)
Secondary complete	04	(12.9)	04	(9.1)
Incomplete high school	02	(6.5)	09	(20.5)
High school	01	(3.2)	03	(6.8)
College education	02	(6.5)	-	-

### Anthropometric parameters

Patients with plantar ulcers had a mean weight of 71.6 ± 11.4 kg and a mean height of 1.62 ± 0.1 m. For patients without plantar ulcers, the mean weight was 67.5 ± 12.3 kg and the mean height was 1.60 ± 0.1 m. Measurements of BMI, AC, TSF, and AMC showed no significant associations with the presence or absence of plantar ulcers (*P* > 0.05; Table 2). Most patients (83.9% with and 79.5% without plantar ulcers) had the MB form of leprosy. No significant difference was observed between the variables cited (*P* = 0.863).

**Table 2:** Distribution of leprosy patients according to anthropometric assessment and association with the presence or absence plantar ulcer

Parameter	With ulcer (*n* = 31)		No ulcer (*n* = 44)		*P value
	*n*	(%)	*n*	(%)	
I MC					
Malnutrition	-	-	01	(2.3)	0.7894
Eutrophic	11	(35.5)	17	(38.6)	
Overweight	14	(45.2)	19	(43.2)	
Obesity	06	(19.4)	07	(15.9)	
CB (cm)					
Malnutrition	11	(35.5)	15	(34.1)	0.8379
Eutrophic	16	(51.6)	26	(59.1)	
Overweight	01	(3.2)	01	(2.3)	
Obesity	03	(9.7)	02	(4.5)	
PCT (mm)					
Malnutrition	05	(16.1)	08	(18.2)	0.5466
Eutrophic	06	(19.4)	09	(20.5)	
Overweight	-	-	02	(4.5)	
Obesity	20	(64.5)	25	(56.8)	
CMB (mm)					
Malnutrition	17	(54.8)	28	(63.6)	0.7708
Eutrophic	13	(41.9)	15	(34.1)	
Obesity	01	(3.2)	01	(2.3)	

*Test G (statistical significance level *P* < 0.05)

When examining the correlation between BMI and the clinical forms of leprosy, we found that 64.3% of patients with PB leprosy and 39.3% of patients with MB leprosy were overweight. Furthermore, 7.1% of patients with PB leprosy and 19.7% of patients with MB leprosy were considered to be obese based on BMI calculations.

Our analysis of AC showed that most patients were eutrophic (64.3% of PB and 54.1% of MB patients). Among patients who were classified as malnourished based on AC, 35.7% had PB leprosy and 34.4% had MB leprosy. A total of 11.5% of patients with MB leprosy were considered to be overweight based on AC.

A nutritional assessment based on TSF measurements indicated that most patients (57.1% with PB leprosy and 60.7% with MB leprosy) were obese. Eutrophy and malnutrition were each present in 21.4% of patients with PB leprosy. However, for patients with MB leprosy, 16.4% were malnourished and 19.7% were eutrophic based on TSF measurements.

AMC was measured to determine lean mass. We found that 57.1% of patients with PB leprosy and 60.7% of patients with MB leprosy were classified as malnourished. An adequate nutritional status was observed in 42.9% of patients with PB leprosy and 36.1% of patients with MB leprosy. No significant associations were found between the anthropometric parameters and clinical forms of leprosy (Table 3).

**Table 3:** Frequency distribution of patients according to the anthropometric data and association with clinical forms

Parameter	Paucibacillar (*n* = 14)		Multibacillar (*n* = 61)		*P value
	*n*	%	*n*	%	
BMI (kg/m^2^)					
Malnutrition	01	(7.1)	-	-	0.0599
Eutrophic	03	(21.4)	25	(41.0)	
Overweight	09	(64.3)	24	(39.3)	
Obesity	01	(7.1)	12	(19.7)	
AC					
Malnutrition	05	(35.7)	21	(34.4)	0.3763
Eutrophic	09	(64.3)	33	(54.1)	
Overweight	-	-	02	(3.3)	
Obesity	-	-	05	(8.2)	
TSF					
Malnutrition	03	(21.4)	10	(16.4)	0.7952
Eutrophic	03	(21.4)	12	(19.7)	
Overweight	-	-	02	(3.3)	
Obesity	08	(57.1)	37	(60.7)	
AMC					
Malnutrition	08	(57.1)	37	(60.7)	0.6108
Eutrophic	06	(42.9)	22	(36.1)	
Obesity	-	-	02	(3.3)	

*BMI* body mass index, *AC* arm circumference, *TSF* triceps skinfold, *AMC* arm muscle circumference

*Test G (significance level *P* < 0.05)

### Biochemical parameters

Serum albumin analysis revealed nutritional depletion of this protein in 25.8% of patients without plantar ulcers and 22.7% of patients with plantar ulcers. Most patients had normal serum levels (74.2% with plantar ulcers and 77.3% without plantar ulcers).

Elevated CRP levels were observed in 51.6% of patients with plantar ulcers and 9.1% of patients without plantar ulcers. Serum levels were normal in 48.4% of patients with plantar ulcers and 90.9% of patients without plantar ulcers. Statistical analysis showed a significant difference between the groups with and without plantar ulcers (*P* < 0.001).

Nutritional depletion of transferrin was observed in 45.2% of patients with plantar ulcers and in 34.1% of patients without plantar ulcers. Transferrin levels were normal in 54.8% and 63.6% of patients with and without plantar ulcers, respectively (Table 4).

**Table 4:** Distribution of frequency and association between the biochemical variables and the occurrence of plantar ulcer

Parameter	With ulcer (*n* = 31)		No ulcer (*n* = 44)		*P* value
	*n*	(%)	*n*	(%)	
Albumin					
Normal	23	(74.2)	34	(77.3)	0.9737
Depletion	08	(25.8)	10	(22.7)	
PCR (mg/dl)					
Normal	15	(48.4)	40	(90.9)	<0.0001*
High	16	(51.6)	04	(9.1)	
Transferrin (mg/dl)					
Normal	17	(54.8)	28	(63.6)	0.5143
Depletion	14	(45.2)	15	(34.1)	

Chi-square / * Fisher’s exact test (level of statistical significance *P* < 0.05). Normal levels: Albumin = 35–55 g/L; PCR = 0.1 mg/dl; Transferrin = 250–380 mg/dl

When examining the correlation between serum albumin levels and the clinical forms of leprosy, we found no differences in the serum levels of this protein in most patients with the PB and MB forms of leprosy. Normal serum levels of CRP were detected in 92.9% of patients with PB leprosy and 68.9% of patients with MB leprosy. Elevated levels of CRP were observed in 7.1% of patients with PB leprosy and 31.1% of patients with MB leprosy.

Analysis of serum transferrin levels showed that 85.7% of patients with PB leprosy and 55.7% of patients with MB leprosy maintained normal levels. Nutritional depletion of transferrin was observed in 14.3% of patients with PB leprosy and 44.3% of patients with MB leprosy. A significant difference in serum transferrin levels was observed between the clinical forms of leprosy (*P* < 0.05; Table 5).

**Table 5:** Distribution of clinical forms of leprosy as the biochemical parameters

Biochemical	Paucibacillar (*n* = 14)		Multibacillar (*n* = 61)		*P* value
	*n*	%	*n*	%	
Albumin (g/dl)					
Normal	13	(92.9)	44	(72.1)	0.0923
Depletion	01	(7.1)	17	(27.9)	
PCR (mg/dl)					
Normal	13	(92.9)	42	(68.9)	0.0595
High	01	(7.1)	19	(31.1)	
Transferrin (mg/dl)					
Normal	12	(85.7)	34	(55.7)	0.0447*
Depletion	2	(14.3)	27	(44.3)	

*Fisher’s exact test (significance level *P* < 0.05%)

### Food intake and protein levels

We examined the frequency of food group intake for all patients. Patients with plantar ulcers reported an average intake of 25.8% red meat and 25.8% eggs, whereas patients without plantar ulcers reported an average intake of 22.7% red meat and 6.8% eggs. Furthermore, a moderate to high intake of salted meat was observed for 54.8% of patients with plantar ulcers and 40.9% without plantar ulcers.

A high intake of whole milk was reported by both patients with plantar ulcers (71.0%) and patients without plantar ulcers (70.5%). With regard to legumes, the consumption of beans predominated, with 67.8% of patients with plantar ulcers and 65.9% of patients without plantar ulcers reporting medium to high intake. No significant association was observed between food intake and plantar ulcers (Table 6).

**Table 6:** Distribution of the frequency of patients with or without plantar ulcers according to consumption of food protein

Food Groups	Consumption	With ulcer (*n* = 31)		No ulcer (*n* = 44)		*P value
		*n*	%	*n*	%	
Meat and eggs						0.6069
Beef	Low	21	67.7	33	75.0	
	Medium	08	25.8	10	22.7	
	High	02	6.5	01	2.3	
Eggs	Low	22	71.0	40	90.9	0.0669
	Medium	08	25.8	03	6.8	
	High	01	3.2	01	2.3	
Beef Jerky	Low	14	45.2	26	59.1	0.4321
	Medium	08	25.8	10	22.7	
	High	09	29.0	08	18.2	
Milk and Dairy Products						
Whole milk	Low	08	25.8	11	25.0	0.9586
	Medium	01	3.2	02	4.5	
	High	22	71.0	31	70.5	
Vegetables						0.4138
Bean	Low	10	32.3	15	34.1	
	Medium	07	22.6	05	11.4	
	High	14	45.2	24	54.5	

*Chi-square test (significance level *P* < 0.05)

## Discussion

In this study, most patients with leprosy (with and without ulcers) were male patients, which is in agreement with the results of Barreto and Salgado [21], Gaschignard et al. [22] and Chaptini and Marshman [23]. However, some countries have reported a higher incidence of the disease among women or no sex difference at all [24–26].

The age groups most frequently affected by the disease are part of the economically active population. Thus, leprosy directly affects patients’ work capacity and social life because of its high potential for disability, which is in agreement with the results reported by Gaschignard et al. [22] and Chaptini and Marshman [23]. With respect to marital status, most leprosy patients had partners. Similar results have been reported by Silva et al. [27] and Guyon et al. [28].

Most patients had no defined occupation. Majority of the patients with plantar ulcers belonged to the retirement/benefit category a finding that can be explained by the disability caused by plantar ulcers, which likely prevents these patients from taking up employment. In a study by Baldan [29], 42% of the participants were actively employed, 26% were retired, 24% were on a leave of absence, and 8% were unemployed. Femina et al. [30] reported that 26.7% of patients had employment difficulties, of which 66.7% were on a leave of absence, 25% had difficulties performing their jobs, and 8.3% were laid off. In studies by Batista et al. [31] and Guyonet et al. [28], there was a predominance of activities that did not require professional qualification. The physical disabilities caused by leprosy thus seem to significantly limit employment opportunities in most areas.

In our study, most patients reported a monthly income between US$ 250.00 and US$ 500.00, as well as an education level of incomplete elementary school. Similar results were reported by Baldan [29], Santos et al. [32], Chaptini and Marsham [23] and Gaschignard et al. [22].

Most patients in our study had MB leprosy, with no statistically significant difference found between clinical forms. Gomes et al. [33] obtained similar results but found a significant difference. However, Santos et al. [32] observed a significant association between the MB form and limitations in activities. This association is probably because MB leprosy is more contagious and highly disabling. Furthermore, a late diagnosis increases the occurrence of physical disabilities.

According to our BMI classification criteria, many of the patients with leprosy, both with and without plantar ulcers, were overweight or obese. Similar results were reported by Chaptini and Marsham [23], Moura et al. [34], and Canicoba et al. [35]. However, Vaz et al. [36] and Khandapani and Mishra [37] reported a malnutrition rate of 57% among Indian patients with leprosy. This difference may be explained by the lower poverty rate in Latin America compared with India, where the frequency of malnutrition is higher.

Our AC analysis showed a larger number of eutrophic patients with leprosy. Despite this, malnutrition was present in 35.5% of patients with plantar ulcers and in 34.1% of patients without plantar ulcers. The frequency of malnutrition was also similar for patients with the PB and MB forms of leprosy. TSF measurement results showed that most patients, both with and without plantar ulcers, and with either PB or MB leprosy, were overweight or obese. A greater percentage of malnourished patients with leprosy were observed in the PB group. Finally, AMC measurements classified most patients, both with and without plantar ulcers, as malnourished. Similar results were obtained for the PB and MB forms of leprosy. Similar results regarding AC have been reported by Montenegro et al. [38]. In that study, TSF evaluation showed equivalent results for malnourished patients and patients with excess peripheral fat. In the evaluation of AMC, 31.9% of patients had low muscle mass.

Serum albumin analysis revealed that 25.8% of patients without plantar ulcers and 22.7% of patients with plantar ulcers exhibited nutritional depletion; however, the serum levels of this protein were normal in most patients with and without plantar ulcers. When serum albumin levels were compared between patients with PB and MB leprosy, most patients did not show changes. In a cross-sectional study conducted by Oliveira et al. [39] of 59 patients with PB and MB leprosy, all patients had an average albumin level of 4.7 ± 0.38 g/dl (reference range: 3.4–5.4 g/dl).

Brugler et al. [40] showed that serum albumin concentration and the presence of a wound were correlated with the risk of malnutrition-related complications. In a prospective, controlled, observational follow-up study of 41 patients with leg ulcers and 43 healthy controls, Legendre et al. [41] reported that hypoalbuminemia and elevated serum CRP levels in patients with ulcers were statistically significant (*P* < 0.001) when compared to the control group.

We observed a reduction in albumin levels in patients with both forms of leprosy, although most of the patients had MB leprosy. This is characteristic of severe malnutrition and may be a consequence of the effects of inflammation associated with inadequate calorie and protein intake in patients with chronic diseases, such as leprosy. Inflammation and malnutrition reduce the concentration of albumin by decreasing its synthesis rate. The cascade of events arising from inflammation induces anorexia and reduces the effective use of dietary protein and energy intake, thus increasing the catabolism of albumin [42].

Our results show significantly higher CRP levels in leprosy patients with plantar ulcers (*P* < 0.0001), most of whom had MB leprosy. In an evaluation of patients with leprosy and a control group of healthy subjects, Dormelles et al. [43] observed significantly higher CRP concentrations in leprosy patients with the lepromatous form (*P* = 0.0001) when compared to the control group. In contrast, Oliveira et al. [39] found high CRP levels in only 3% of 59 patients with the PB and MB forms of leprosy. Hyun et al. [44] investigated the significance of serum CRP levels in leprosy patients with plantar ulcers. Of 20 patients with plantar ulcers, 4 patients had elevated CRP levels, 1 patient had a clinically evident infection, and 3 patients had erythematous skin of uncertain cause surrounding the ulcers. No increase in CRP levels was observed in patients without ulcers.

The Prognostic Inflammatory and Nutritional Index (PINI) is calculated as CRP + alpha-1-glycoprotein acid/albumin + transthyretin, which is a combination of two positive acute-phase proteins (CRP and alpha-1-glycoprotein acid) and two negative acute-phase proteins (albumin and transthyretin). In an attempt to simplify PINI, Shioya et al. [45] replaced it with the simpler and less expensive CRP/albumin ratio without a loss of power or sensitivity for determining the risk of complications. Furthermore, the authors observed that albumin, transferrin, and prealbumin showed a significant negative correlation with CRP in a group of patients with different degrees of morbidity and mortality.

In our study, nutritional deficiencies detected by the measurement of transferrin were greater in patients with plantar ulcers. A significant difference (*P* < 0.05) was shown for patients with MB leprosy. Transferrin depletion also affected more patients than hypoalbuminemia or elevated CRP levels. Transferrin has a shorter half-life than albumin. Furthermore, although it is not a specific marker of protein malnutrition because its serum concentration is affected by factors such as kidney and liver disease, iron deficiency, and cancer, this protein responds faster to nutritional therapy than albumin. This finding also was reported by Gurski et al. [46], who measured serum transferrin before and after nutritional support and demonstrated a significant increase in this protein after the administration of nutrition therapy.

The consumption of protein was low for most patients both with and without plantar ulcers, with no significant difference between the food groups studied or between the presence and absence of plantar ulcers. Different results were reported by Chaptini and Marshman [25], who observed protein intake above the recommended daily requirement, especially in men. Similar results were reported by Montenegro et al. [38], who found milk, eggs, chicken, and beans to be the most consumed foods by patients with leprosy. The conflicting results of these studies may be due to the socioeconomic conditions of our study population, who had limited abilities to purchase adequate food supplies.

## Conclusions

In our study, most leprosy patients with plantar ulcers had normal serum levels of albumin and transferrin, but high serum CRP levels, which indicate the existence of an inflammatory process. These results suggest the need to monitor patients with leprosy to prevent the occurrence of plantar ulcers and to provide adequate treatment for patients with existing plantar ulcers.

## Additional file

Additional file 1: Multilingual abstracts in the five official working languages of the United Nations. (PDF 614 kb)

## Abbreviations

AC: Arm circumference
AMC: Arm muscle circumference
BMI: Body mass index
CRP: C-reactive protein
MB: Multibacillary leprosy
PB: Paucibacillary leprosy
PUD: Plantar perforating disease
TSF: Triceps skinfold
WHO: World health organization
