# Supplementary material for: Protein profile of leprosy patients with plantar ulcers from the Eastern Amazon region
Source: Infect Dis Poverty. 2017 Sep 4;6:105. doi: 10.1186/s40249-017-0318-y (PMC5582394; doi:10.1186/s40249-017-0318-y)
Supplement: Additional file 1: — Multilingual abstracts in the five official working languages of the United Nations. (PDF 614 kb) [file 40249_2017_318_MOESM1_ESM.pdf]

Translation of the abstract into the five official working languages of the United Nations

**بروفايل البروتين لمرضي الجذام المصابين بقرح أخمصيه من منطقة الأمازون الشرقية**

مارينيا بورتو دي أوليفيرا, خورخي رودريجيز دي سوزا, رافاييل سيلفا دي أراوخو, تينارا ليلي دي سوزا أراو, خواريز أنتونيو سيمويس كواريسما

**الملخص**

**الخلفية:** تعتبر الدراسات التي تحقق في الحالة الغذائية لمرضي الجذام والقرح الأخمصيه شحيحه ولذلك فإن الهدف من الدراسة الحالية هو وصف بروفايل البروتين لمرضي الجذام المصابين بقرح أخمصيه في منطقة الأمازون الشرقية.

**الأساليب:** تم إنشاء نموذج سجل حالة لعدد 75 مريض بالجذام (منهم 31 مريض مصابين بقرحة أخمصيه و44 مريض غير مصابين بها) يحتوي علي البيانات التالية: الخصائص الاجتماعية والحالة السريرية للجذام ووجود وعدم وجود قرح أخمصيه والتقييم الغذائي بإستخدام قياسات بشرية تتكون من قياس مؤشر كتلة الجسم من خلال محيط الذراع و محيط عضلة العضد وثنية جلد العضلة ثلاثية الرؤوس كما تم أيضا قياس مستويات الزلال في الدم والترانسفيرين (بيتاغلوبولين ينقل الحديد في البلازما) وبروتين سي التفاعلي وتم الحصول علي بيانات إستهلاك البروتين بإستخدام إستبيان معدل استهلاك الغذاء.

**النتائج:** كانت القرحة الأخمصيه أغلب ما تكون في المرضي الذكور (بنسبة 67.7٪) والمرضي الذين تتراوح أعمارهم ما بين 40 و 49 عام (mean ± SD: 47.3 ± 8.0 years) وكذلك المرضي الذين يحصلون علي دخل شهري قدره 300 أو 600 دولار, وكان متوسط وزن المرضي هو 71.6 كيلوجرام بإنحراف معياري قيمته 11.4 كيلوجرام ومتوسط الطول كان 1.62 متر بإنحراف معياري قيمته 0.1 متر, كانت مستويات بروتين سي التفاعلي عالية في نسبة 51.6٪ من مرضي الجذام المصابين بقرحة أخمصيه ونسبة 9.1٪ في المرضي الغير مصابين بالقرحة الاخمصيه ($P < 0.001$), وتم ملاحظة نفاد الترانسفيرين الغذائي في نسبة 14.3٪ من مرضي الجذام قليل العصيات ونسبة 44.3٪ من مرضي الجذام متعدد العصيات ($P = 0.0447$), وكان معظم المرضي لديهم نسب طبيعية من الزلال (بنسبة 74.2٪ من المرضي المصابين بالقرح الأخمصيه ونسبة 77.3٪ من المرضي الغير مصابين بها).

**الإستنتاجات:** كان معظم مرضي الجذام المصابين بقرح أخمصيه لديهم نسب طبيعية من الزلال والترانسفيرين ومستويات بروتين سي التفاعلي ويعد ذلك مؤشرا علي وجود عملية إلتهاب, وتشير النتائج التي توصلنا إليها إلى الحاجة لرصد مرضي الجذام لمنع حدوث قرح أخمصيه ولتقديم العلاج المناسب للمرضي المصابين فعليا بقرح أخمصيه.

Translated from English version into Arabic by Mohamed Habib

**东亚马逊地区患有足底溃疡的麻风患者的蛋白质谱研究**

Marineia Porto de Oliveira, Jorge Rodrigues de Sousa,Rafael Silva de Araujo, Tinara Leila de Sousa Aarão, Juarez Antonio Simões Quaresma

**摘要**

**引言：**目前关于麻风和足底溃疡患者营养状况方面的研究较少。因此，本研究的目的是描述东亚马逊地区患有足底溃疡的麻风患者的蛋白质谱。

**方法：**本研究建立了一个包含 75 个麻风患者的病历记录表（31 例患足底溃疡和 44 无足底溃疡），包含以下信息：社会人口学特征，麻风的临床形式，是否患有足底溃疡，以及运用人体测量学评估的营养状况指标，包括体重指数、上臂围、上臂肌围和三头肌皮褶厚度。测定了血清白蛋白、转铁蛋白和 C-反应蛋白（CRP）的水平。使用食物频率问卷获得了有关蛋白质摄入量的数据。

**结果：**结果显示，男性患者（67.7%）、年龄在 40 – 49 岁之间的患者（平均±SD：47.3±8 岁）以及接受 300 美元或 600 美元治疗的患者的足底溃疡发生率较高。患者的平均体重和身高分别为 71.6 ± 11.4 kg 和 1.62 ± 0.1 m。51.6%的患有足底溃疡的麻风患者检测到高水平的 CRP，而无足底溃疡的麻风患者中仅有 9.1%检测到高水平 CRP（$P$＜0.001）。14.3%的少菌

型麻风患者和 44.3%的多菌型麻风患者体内发现存在转铁蛋白营养缺乏（P＝0.0447）。大多数患者血清白蛋白水平正常（伴足底溃疡的患者中 74.2%正常，无足底溃疡的患者中 77.3%正常）。
**结论：**大多数患有足底溃疡的麻风患者体内的血清白蛋白和转铁蛋白水平正常，而 CRP 水平较高，这表明有炎症存在。我们的研究结果表明，有必要对麻风患者进行监测，以防止其出现足底溃疡，并为已患有足底溃疡的患者提供适当的治疗。

Translated from English version into Arabic by Zhou Guan, edited by Pin Yang

**Profil protéique chez les patients atteints de la lèpre avec ulcères plantaires de la region Est de l'Amazonie**

Marineia Porto de Oliveira, Jorge Rodrigues de Sousa,Rafael Silva de Araujo, Tinara Leila de Sousa Aarão, Juarez Antonio Simões Quaresma

**Résumé**
**Contexte:** Les études sur l'état nutritionnel des patients atteints de la lèpre et les ulcères plantaires sont rares. L'objectif de cette étude était de décrire le profil protéique chez les patients atteints de la lèpre avec ulcères plantaires de la region Est de l'Amazonie.
**Méthodes:** Une étude de cas a été créé pour 75 patients atteints de la lèpre (31 avec ulcères plantaires et 44 sans ulcères plantaires) avec les données suivantes: caractéristiques sociodémographiques, forme clinique de la lèpre, présence ou absence d'ulcères plantaires et évaluation nutritionnelle basée sur l'anthropométrie composée de la mesure de l'indice de masse corporelle, de la circonférence du bras, de la circonférence du muscle du bras, du pli cutané du triceps. Les taux d'albumine dans le sang, de transferrine, et de protéine C-réactive (CRP) ont aussi été mesurés. Les données relatives à l'apport en protéines ont été obtenues à l'aide d'un questionnaire sur la fréquence de consommation.
**Résultats:** Les ulcères plantaires sont survenus plus fréquemment chez les patients de sexe masculin (67,7%), les patients âgés de 40 à 49 ans (moyenne ± SD: 47,3 ± 8,0 ans), et les patients recevant 300 ou 600 usd (71,0%). Le poids et la taille moyens des patients étaient 71,6 ± 11,4 kg et 1,62 ± 0,1 m respectivement. Des taux élevés de CRP ont été détectés chez 51,6% de patients atteints de la lèpre avec ulcères plantaires, et chez 9,1% de patients atteints de la lèpre sans ulcères plantaires (p < 0001). La perte nutritionnelle de transferrine est observée chez 14,3% de patients atteints de la lèpre paucibacillaire et 44,3% de patients atteints de la lèpre multibacillaire (p = 0.0447). La plupart des patients avaient des taux normaux d'albumine sérique (74,2% chez des ulcères plantaires et 77,3% chez des sans ulcères plantaires).
**Conclusion:** La plupart des patients atteints de la lèpre ave ulcères plantaires ont des taux normaux d'albumine sérique et de transferrine, et des taux élevés de CRP, ce qui indique la présence d'un processus inflammatoire. Nos résultats suggèrent la nécessité de surveiller les patients atteints de la lèpre afin de prévenir l'apparition des ulcères plantaires et de fournir un traitement adéquat pour les patients avec les ulcères plantaires.

Translated from English version into French Ephrem De Vera

**Показатели белка у больных проказой с подошвенными язвами, живущих в Восточной Амазонии.**

Marineia Porto de Oliveira, Jorge Rodrigues de Sousa,Rafael Silva de Araujo, Tinara Leila de Sousa Aarão, Juarez Antonio Simões Quaresma

**Аннотация**

**Введение:** Исследования, изучающие состояние питания у пациентов с проказой (лепрой) и подошвенными язвами, довольно редки. Исходя из этого, предмет данного исследования заключался в описании показателя белка у больных проказой с подошвенными язвами, живущих в Восточной Амазонии.
**Методы исследования:** Для 75 пациентов с проказой (31 из которых имели подошвенные язвы, 44 не имели таковых) была создана индивидуальная регистрационная форма, которая содержала в себе следующую информацию: социально-демографическая характеристика, клиническая форма проказы, наличие или отсутствие подошвенных язв, оценка состояния питания при помощи антропометрии, заключающейся в расчете индекса массы тела, замере окружности плеча, окружности мышц плеча, и кожной складки трицепса. Так же были проведены замеры уровня альбумина, трансферрина и С-реактивного белка (CRP) в крови. Данные, касающиеся белков были получены с использованием опросника частоты потребления различных пищевых продуктов.
**Результаты исследования:** Подошвенные язвы более часто наблюдались у мужского пола (67.7%), у пациентов в возрасте 40-49 лет (± стандартное отклонение: 47.3 ± 8.0 лет), и у пациентов зарабатывающих 300 или 600 долларов США (71.0%). Средний вес и рост пациентов достигали 71.6 ± 11.4 кг и 1.62 ± 0.1 м, соответственно. Высокий уровень С-реактивного белка обнаружен у 51.6% больных проказой с подошвенными язвами и только у 9.1% больных без таковых ($P < 0.001$). Уменьшение уровня трансферрина в рационе питания было обнаружено у 14.3% больных олигобациллярной проказой и 44.3% больных мультибациллярной проказой ($P = 0.0447$). Большинство пациентов имело предельно допустимый уровень сывороточного альбумина (74.2% с подошвенными язвами and 77.3% без таковых).
**Выводы:** Большинство больных проказой с подошвенными язвами имеют допустимый уровень сывороточного альбумина, трансферрина и высокий уровень С-реактивного белка, что указывает на наличие воспалительного процесса. Наши данные указывают на необходимость контроля за состоянием больных проказой для предотвращения случаев возникновения подошвенных язв и обеспечения надлежащего лечения пациентов с существующими подошвенными язвами.

Translated from English version into French by Hao-Qi Zhang

**Perfil proteico de pacientes con lepra con úlceras plantares de la región Amazónica Oriental**

Marineia Porto de Oliveira, Jorge Rodrigues de Sousa, Rafael Silva de Araujo, Tinara Leila de Sousa Aarão, Juarez Antonio Simões Quaresma

**Abstracto**
**Trasfondo:** Los estudios que investigan el estado nutricional de pacientes con lepra y úlceras plantares son escasos. Por lo tanto, el objetivo de este estudio fue describir el perfil proteico de pacientes con lepra, con úlceras plantares de la región oriental Amazónica.
**Métodos**: Un récord de casos fue creado para 75 pacientes con lepra (31 con úlceras plantares y 44 sin úlceras plantares) con los siguientes datos: características sociodemográficas, forma clínica de la lepra, presencia o ausencia de úlceras plantares y la evaluación nutricional utilizando la antropometría, la cual consiste en la medición del índice de masa corporal, la circunferencia del brazo y del músculo y los dobleces en la piel del tríceps. También se midieron los niveles de albúmina en sangre, transferrina y proteína C reactiva (CRP, por sus siglas en inglés). Los datos relativos a la ingesta de proteínas se obtuvieron utilizando un Cuestionario de Frecuencia Alimentaria.
**Resultados:** Las úlceras plantares ocurrieron con mayor frecuencia en pacientes masculinos (67.7%), pacientes de 40 a 49 años (media ± desviación estándar: 47.3 ± 8.0 años) y pacientes que recibieron 300 ó 600 USD (71.0%). El promedio de peso y altura de los pacientes fue de 71.6 ± 11.4 kg y 1.62 ± 0.1 m, respectivamente. Se detectaron altos niveles de CRP en el 51.6% de los pacientes con lepra con úlceras plantares y sólo en el 9.1% de los pacientes sin úlceras plantares ($P$ <0.001). Una falta nutricional de transferrina se observó en el 14.3% de los

pacientes con lepra paucibacilar y en 44.3% de los pacientes con lepra multibacilar ($P$ = 0.0447). La mayoría de los pacientes tenían niveles normales de albúmina en suero (74.2% con úlceras plantares y 77.3% sin úlceras plantares).
**Conclusiones:** La mayoría de los pacientes con lepra y con úlceras plantares tienen niveles normales de albumina y transferrina y altos niveles de CRP en suero, lo que indica la presencia de un proceso inflamatorio. Nuestros hallazgos sugieren la necesidad de monitorear los pacientes con lepra para prevenir la aparición de úlceras plantares y para proveer un tratamiento adecuado para los pacientes existentes con úlceras plantares.

Translated from English version into French by Laura C Vicente Rodriguez
